# Healthcare Cost Reductions after Moving into a Wet Nursing Home Stay—A Case Series

**DOI:** 10.3390/geriatrics2040031

**Published:** 2017-09-26

**Authors:** Henrik Thiesen, Lene Tanderup, Bodil Stavad, Morten Hesse

**Affiliations:** Health Team for the Homeless, Municipality of Copenhagen, Copenhagen S 2300, Denmark; GG63@sof.kk.dk (H.T.); LD52@sof.kk.dk (L.T.); LD53@sof.kk.dk (B.S.)

**Keywords:** alcohol dependence, dementia, externalizing behavior problems, harm reduction, healthcare costs

## Abstract

Serious alcohol dependence is associated with high healthcare costs, especially when patients have chronic problems with alcohol, dementia and exhibit externalizing behavior. One option is to offer a wet nursing home for seriously ill patients for whom abstinence from alcohol is not a feasible option. In this case series, we present the healthcare costs 18 months before moving into a “wet nursing home”, and in the first 18 months of their stay, for three cases, one with low needs of care, one with medium needs, and one with high needs. Results: for all three patients, hospital costs were reduced by between 83.7 and 97.9% for patients with dementia, externalizing behavior, and chronic alcohol problems, a wet nursing home can produce substantial cost reductions in other parts of the healthcare sector.

## 1. Introduction

Although the course of alcohol problems over a lifetime is highly heterogeneous, a significant proportion of people with alcohol problems experience serious problems well into old age [1]. Prolonged heavy drinking is associated with considerable health problems [2,3], which in turn leads heavy drinkers to seek a considerable amount of healthcare services [4].

At the same time, alcohol problems, as well as many of the problems associated with it, may lead patients to decline healthcare until the situation becomes unbearable or dangerous, which ends up requiring more intensive treatment for longer periods. Conversely, general healthcare systems may try to avoid admitting patients with current alcohol problems to treatment, or may release patients prematurely due to behavioral problems in medical settings.

This state of affairs has in recent decades led to the view that treatment for substance use disorders should adopt a “chronic care” perspective [5]. Under such a perspective, clinicians need to adjust services and interventions to the course of the illness, rather than assume that the condition can be managed without regard to the duration of illness, complications, and other factors such as the age of the patient or the socioeconomic status of the patient.

In general, attending low-threshold services can improve functioning in socially marginalized alcohol-dependent people, even without any requirement that they decrease their use of substances [6]. Some experts within the broader addictions field refer to such pragmatic strategies as “harm reduction” [7]. This approach entails reaching patients where they are, rather than assuming an ideal state for patients, and goes beyond treatment, into the broader management of risk, including legislation, outreach programs, patient education, and providing services in a non-judgmental fashion.

There is some evidence that shelter-based administration of alcohol can help seriously alcohol-dependent homeless people cope better and function better [8]. This suggests that some form of “supported controlled drinking” can help a substantial proportion of seriously alcohol-addicted people function better and obtain a better quality of life.

For severely alcohol-dependent patients who are older or physically impaired, the next logical step is the wet nursing home. The wet nursing home is the nursing home for alcohol-dependent people who need an amount of care that cannot be provided in one’s own home, but whose alcohol use and associated problems is an obstacle for living in a general nursing home.

## 2. Materials and Methods

### 2.1. Setting

The setting is “E-huset”, a wet nursing home for patients with alcohol problems, dementia, and externalizing behavior. Patients referred to the nursing home must be in contact with healthcare, or have a home nurse provided by social services. The patient must also have a severe alcohol use disorder as evaluated by the referring help system, and associated behavioral problems. The nursing home does not require residents to become alcohol abstinent or to reduce their alcohol use. If the resident stops drinking he or she will be referred to an ordinary nursing home. There are no restrictions on alcohol use, but staff members consistently attempt to direct the use into lower-percent alcohol beverages and reduce episodes of binge-drinking.

In most other ways, the home provides the care and activities of a typical nursing home, although the residents are considerably younger than in the typical nursing home.

During the months from September to December of 2006, three cases were selected by staff at the home to assess cost reductions in healthcare, to represent a “high needs”, “medium needs” and “low needs” case. No data was collected on healthcare utilization prior to selecting the cases. The cases were selected to represent varying degrees of need for care, and to be able to provide a formal consent.

Healthcare costs for inpatient treatment were estimated based on data from the patients’ medical files. Additional information about the costs of particular types of healthcare services was sought from regional hospital services. Outpatient treatment and treatment in emergency rooms are registered but it has not been possible to estimate the price of these services. Thus, the hospital costs in the following cases are based exclusively on inpatient services.

Changes in costs were estimated as the difference between the total costs of inpatient services for the patient in the eighteen months prior to moving in to the nursing home minus the total costs of inpatient services during the eighteen months following admission to the home. Further, the types of services used by each patient are described, along with information about the residents’ medical histories.

### 2.2. Ethics

Consent to participate was sought with the patient and presented to the health authorities.

## 3. Results

The costs before and after moving into the home are summarized in Table 1. The following provides a narrative description of the three cases.

### 3.1. Case 1. Low Level of Need

Case 1 was a 60-year-old woman. She was raised in a coastal area in a small town, in what is described as a well-functioning family, and has been trained as a healthcare assistant. Prior to moving into the nursing home, she had been married and had two adult children, with whom she had no contact. She had gradually increased her consumption of alcohol over the years, and in spite of several contacts with outpatient services for alcohol dependence, her drinking had steadily increased. After losing her job, she became increasingly socially isolated. Prior to moving into the nursing home, her home nurses visited several times per day, and they often found her in severe withdrawal, occasionally convulsing. She was underweight and incontinent. Her apartment was untidy and rarely cleaned, smelled of urine and feces and evinced her lack of personal hygiene. She was depressed and talked about suicide.

After moving into the home, she gradually became stable, and was able to manage her personal hygiene with minimal assistance. She ate at meals and began to look better. She was still drinking, but at a level that did not cause problems with other residents. Occasionally, she drank heavily for 1–2 weeks. Her contact with other residents and staff stabilized, and she participated in simple practical activities. She seemed less anxious, and did not go through serious withdrawal.

During the 18-month period before moving into the nursing home, she had been hospitalized nine times for periods ranging from 1 to 19 days; in total, she spent 43 days in hospital, had one outpatient visit and several ER visits. The total cost of her hospital-based care was estimated to be 154,649 DKK (20,798.74 Euros).

After moving into the nursing home, she was admitted to inpatient treatment on two occasions for a total of 4 days. The total cost was 25,226 DKK (3392.64 Euros). She also had two visits at an emergency room (ER) and four outpatient visits.

### 3.2. Case 2. High Need of Care

Case 2 was a 62-year-old man. He had been raised in what is described as a well-functioning family. He had no formal training, but had been working for most of his life as an unskilled worker. He had been married three times, and had three adult children, with whom he had no contact. After his last divorce, his consumption of alcohol increased rapidly, causing him to lose his job. Because of alcohol problems and depression, he was repeatedly hospitalized. After an acute cerebral infarction at age 60, he was left with brain damage that rendered him unable to take care of himself. Apart from alcoholic drinks, he started drinking chlorine, denatured alcohol and toilet cleaner. He was described as depressed, lonely and completely without initiative.

After moving into the nursing home, he started to eat, consumed alcohol in an acceptable manner, and his health condition improved considerably. He also made contact with the other residents and staff, reducing his loneliness.

During the 18-month period prior to moving into the nursing home, he was admitted to inpatient wards eleven times, and spent a total of 237 days in hospital. The total cost of these hospitalizations is 1,023,830 DKK (137,694.90 Euros). Due to aggressive/psychotic behavior during intensive care, he also had to be closely supervised by extra staff, but the costs associated with this extra staff could not be estimated by the unit. Further, he had four emergency room visits, one psychiatric emergency and eight outpatient visits.

During his first 18 months in the nursing home, he was hospitalized once for three days. The total healthcare cost was 21,564 DKK (2900.57 Euros). In that period, he had two outpatient visits and no ER visits.

### 3.3. Case 3. Medium Level of Severity

Case 3 was a 70-year-old man, who had a diverse history of employment, including military service, working as a plumber, and running his own business. He had been married four times and had two daughters. After his fourth divorce, he stated that he intended to drink himself to death. By age 50, he obtained a disability pension due to rheumatism, and developed a serious prescription opioid dependence. He would increasingly leave his home and walk around drinking until he would pass out on a bench or in a park. He was unable to cook meals for himself, and repeatedly forgot to turn off his stove. He had a number of somatic complaints, and asked doctors and nurses for painkillers. Additionally, he had serious financial problems, and was often aggressive and dissatisfied.

After moving into the wet nursing home, he became able to manage his personal hygiene, and made and maintained contact with his sister. His response to pain medication improved, and he appeared to be satisfied with living in the home. He continued to drink, but was almost never seen intoxicated.

In the 18 months prior to moving into the home, he had been hospitalized nine times for a total of 77 days, had one ER visit and 5 outpatient visits. The total cost was 328,579 DKK (44,190.59 Euros). After moving into the home, he was hospitalized once for two days, and had three visits to general ER and four outpatient visits. The total cost of inpatient care during this period was 9458 DKK (1273 Euros).

## 4. Discussion

This case series illustrates the clinical value and the potential cost-benefit of the implementation of the wet nursing home. Given the high amount of healthcare services needed for these patients, the implementation of wet nursing homes is likely to reduce healthcare costs for such patients considerably.

Some important limitations must be acknowledged. First, the absence of a control group means that the patients in the study may have been at a lifetime peak level of healthcare costs when they first moved into the nursing home, and that their level of healthcare service use would have declined regardless of whether they had stayed in their homes or not. We consider this a possibility, but given the serious nature of their alcohol problems and other health issues, we doubt very much that the decline would be comparable to a similarly affected control group outside a specialized nursing home. Also, we did not have an alcohol-free alternative to compare with the wet nursing home. It is uncertain whether the subjects in our study would have been willing to move into a nursing home where they were required to be abstinent or to never be intoxicated, or if they could have complied with those rules. However, our experience from other settings is that a general alcohol-abstinence rule for severely demented people with addiction is an object of constant conflict and results in much less control of drinking.

However, regardless of whether such an alternative would have been feasible for this group of patients, the main focus of this case series was not to assess reductions in alcohol consumption for elderly heavy drinkers who were admitted to a wet nursing home. The significant finding is that the subjects improved considerably in several areas although they were all allowed to continue consumption of alcohol.

A further limitation is that we did not have access to standardized measures of the residents’ degree of dementia, such as the Mini Mental State Examination [9], or to measures of the severity of their alcohol problems.

Future studies should test the wet nursing home model against other forms of care, including continuing care in the home or traditional nursing homes. If it is not feasible to conduct randomized controlled trials, residents in new nursing homes can be compared to historical control groups or to residents in nursing homes outside of the uptake area of the wet nursing home.

Additionally, future research could look into the social costs associated with people with severe alcohol dependence, including social services costs and impact on others, such as neighbors, adult children, and others.

## 5. Conclusions

This case series illustrates how in a wet nursing home, patients with severe alcohol problems can be moved from unacceptable living conditions to conditions that are stable, where immediate risks can be removed from the patients, and where the patients gain access to social contacts. Alcohol harm reduction is potentially important in the context of the most severely affected patients with alcohol use disorders. These targeted services for long-term alcohol users with dementia causes a significant rise in quality of life, just as nursing homes serving the needs of Alzheimer patients elevate the general quality of life for those patients. Another important finding, for policy makers in particular, is that this targeted service also produces a very significant reduction in healthcare costs (Table 1).

## Figures and Tables

**Table 1:** Costs before and after.

Case #	Hospital Costs before Admission (DKK)	Hospital Costs after Admission (DKK)	Savings Related to Pre-Admission Costs (%)
Case 1 (low level need)	154,649	25,226	83.7
Case 2 (high level need)	1,023,830	21,564	97.9
Case 3 (intermediate level need)	328,579	9458	97.1
